# Identification of Putative Equilibrative Nucleoside Transporter Inhibitors Through Dual-Pharmacophore Virtual Screening and Validation in a Gemcitabine-Based Cell Assay

**DOI:** 10.3390/molecules31081293

**Published:** 2026-04-15

**Authors:** Sedra Kremesh, Azza Ramadan, Sedq Ahmad Moutraji, Shaima Hasan, Radwa E. Mahgoub, Imogen R. Coe, Nour Sammani, Lama Abuamer, Noor Atatreh, Mohammad A. Ghattas

**Affiliations:** 1 College of Pharmacy, Al Ain University, Abu Dhabi 64141, United Arab Emirates; 2 AAU Health and Biomedical Research Center, Al Ain University, Abu Dhabi 112612, United Arab Emirates; 3 Institute for Biomedical Engineering, Science and Technology (iBEST), Toronto, ON M5B 1T8, Canada; 4 Department of Chemistry and Biology, Toronto Metropolitan University, Toronto, ON M5B 2K3, Canada

**Keywords:** hENT, hENT inhibitors, gemcitabine, cancer cell lines, MTT assay, virtual screening, pharmacophore modeling, drug discovery

## Abstract

Pharmacological inhibition of the nucleoside transporter hENT1 is a promising therapeutic target across a range of diseases, including cardiovascular disorders, neurodegenerative conditions, and cancer. However, current inhibitors lack drug-like properties, necessitating the development of new inhibitors with improved pharmacological profiles. We employed a dual-pharmacophore virtual screening protocol to identify putative hENT1 inhibitors from a library of over 2 million compounds, followed by structure-based molecular docking. To validate the inhibition effect of the lead compounds, we established a functional assay using gemcitabine (GEM)-induced cytotoxicity as a readout of hENT transport activity using eight cancer cell lines. H292 was the optimal cancer cell line for the validation assay based on its high GEM sensitivity (IC50 = 28 nM) and the concentration-dependent cytotoxicity inhibition of the reference inhibitor NBTI, a hENT1 inhibitor. Of the 19 candidate compounds, two leads (compounds 2 and 3) demonstrated potency comparable to NBTI, increasing GEM IC50 values by 2.2- and 2.9-fold at 5 µM, respectively. Both compounds were non-cytotoxic to normal fibroblasts, exhibited favorable ADME properties, displayed superior docking scores of −12.63 and −12.49 kcal/mol compared to NBTI (−9.06 kcal/mol), and displayed a novel vertical binding orientation within the hENT1 binding pocket distinct from NBTI’s horizontal mode. This study established a validated non-radioactive, gemcitabine-based functional assay for hENT inhibitor discovery and identified two putative inhibitors with therapeutic potential for cancer chemosensitization, pain management, and cardio- and neuroprotection. The non-radioactive functional assay overcomes the limitations of traditional radiolabeled methods, enabling scalable, broader screening applications.

## 1. Introduction

Equilibrative nucleoside transporters (ENTs, SLC29) are cellular membrane-anchored proteins that mediate nucleoside transport across the cell membrane [1]. ENTs are responsible for the transport of nucleosides via energy-independent, sodium-independent uniport down their concentration gradient [2,3,4], a function crucial in cells that lack the de novo purine synthesis pathway, and thus serve as transporters of metabolic precursors for DNA and RNA synthesis and energy metabolism [1,5]. Pharmacologically, ENTs play a critical role in determining the efficacy of many FDA- or EMA-approved drugs [1], including those used in cancer therapies, such as gemcitabine, and antiviral agents, such as ribavirin [6,7].

There are four members of the human ENT family (hENT1–4), of which hENT1 is the best characterized and the major nucleoside transporter in plasma membranes [2,3,4]. hENT1 is expressed in almost all tissue types, with varying abundance, and shows the highest expression in erythrocytes, the vascular endothelium, the gastrointestinal tract, neurons, and glial cells [1,4,8,9].

In animals, hENT1 has been shown to be overexpressed after cerebral infarction or occlusion, and hENT1 inhibition prevents neuronal apoptosis and improves neurological function [9]. In clinical trials of the hENT1 inhibitor draflazine, treatment showed promising results in patients with unstable coronary disease, attributed to increased extracellular adenosine concentration, enhanced signaling via adenosine receptors, vasodilation, and reduced ischemia-induced tissue damage [10,11,12]. ENT1 inhibitors were also found to be neuroprotective in Huntington’s disease, Alzheimer’s disease, and epilepsy due to modulation of adenosine homeostasis [13,14,15]. In oncology, the administration of hENT1 inhibitors in combination with anticancer nucleoside drugs transported by other nucleoside transporters has been proposed to enhance the effects of nucleoside drugs by preventing cellular efflux and reducing the development of drug resistance [16]. For example, CNX-774, an ENT1 inhibitor, increased cell sensitivity to the chemotherapeutic agent brequinar and overcame resistance in pancreatic ductal adenocarcinoma by reducing uridine uptake and intracellular nucleotide pools, and inhibition of ENT1/2 enhanced the effectiveness of BQ in colon and pancreatic cancer cells [16,17].

Studies suggest that ENTs have poor tolerance for chemical diversity in the interactions that lead to high-affinity ligand binding [1]. The highly specific and potent hENT1 inhibitor 4-nitrobenzylthioinosine (NBTI) lacks drug-like properties, being highly polar and having negligible oral bioavailability [18]. Its polar nature prevents its passage into the hydrophobic lipid bilayer, hindering intestinal absorption and preventing its crossing of the blood–brain barrier, thereby limiting its use as a CNS drug [18]. NBTI-mediated off-target effects on the cardiovascular system, hepatic toxicity, and limited brain penetration have been reported [18,19], and dipyridamole, another important hENT1 inhibitor, interacts with α_1_-acid glycoprotein, thereby reducing its effectiveness [20]. Thus, new selective hENT1 inhibitors and clinically relevant drugs are urgently needed.

Previous computational methodologies have been applied for new ENT inhibitor discovery, though with varying approaches. Grixti et al. [21] identified compounds affecting gemcitabine cytotoxicity using cheminformatics-based selection and validated them with a gemcitabine-based functional assay. Li et al. [22] utilized molecular docking combined with SAR-driven synthesis based on a 4-((4-(2-fluorophenyl)piperazin-1-yl)methyl)-6-imino-N-(naphthalen-2-yl)-1,3,5-triazin-2-amine (FPMINT) template and validated their analogs through [^3^H]uridine uptake assays. Dilweg et al. [23] employed scaffold-based design and molecular docking to design and synthesize dilazep derivatives, validating them using [^3^H]NBTI displacement radioligand binding assays. More recently, Zhang [24] conducted an in silico study using molecular docking which resulted in a selection of candidate compounds.

A major limitation in transporter inhibitor discovery is the radiolabeled substrate uptake/transport assay, considered the gold standard for measuring transporter affinity and kinetics [25,26], as the use of radioisotopes makes the assay hazardous, labor-intensive, and costly, hindering the identification of new hENT1 inhibitors.

The primary innovation of this study is the development and validation of a safer and more practical alternative to radioligand-based screening. Specifically, we designed a cytotoxicity-based assay utilizing hENT-expressing cancer cell lines, with gemcitabine transport serving as a functional indicator of transporter activity. This assay eliminates safety hazards associated with radiolabeled substrates, such as [^3^H]-uridine, [^3^H]-NBMPR, and [^3^H] 2-chloroadenosine; reduces assay costs; and enhances scalability for high-throughput screening of hENT inhibitors. Additionally, our systematic multi-cell-line screening approach established an optimal cell-based system for functional validation and enabled more reliable and comprehensive assessment of putative inhibitor activity.

We applied this validated assay to test computationally designed hENT1 inhibitors, where a ligand-based approach employing a dual-pharmacophore virtual screening strategy was applied to screen over 2 million compounds, which led to the identification of two putative hENT inhibitors.

## 2. Results

### 2.1. Identification of 19 hENT1 Inhibitor Compounds Through Virtual Screening

**Figure 1:**
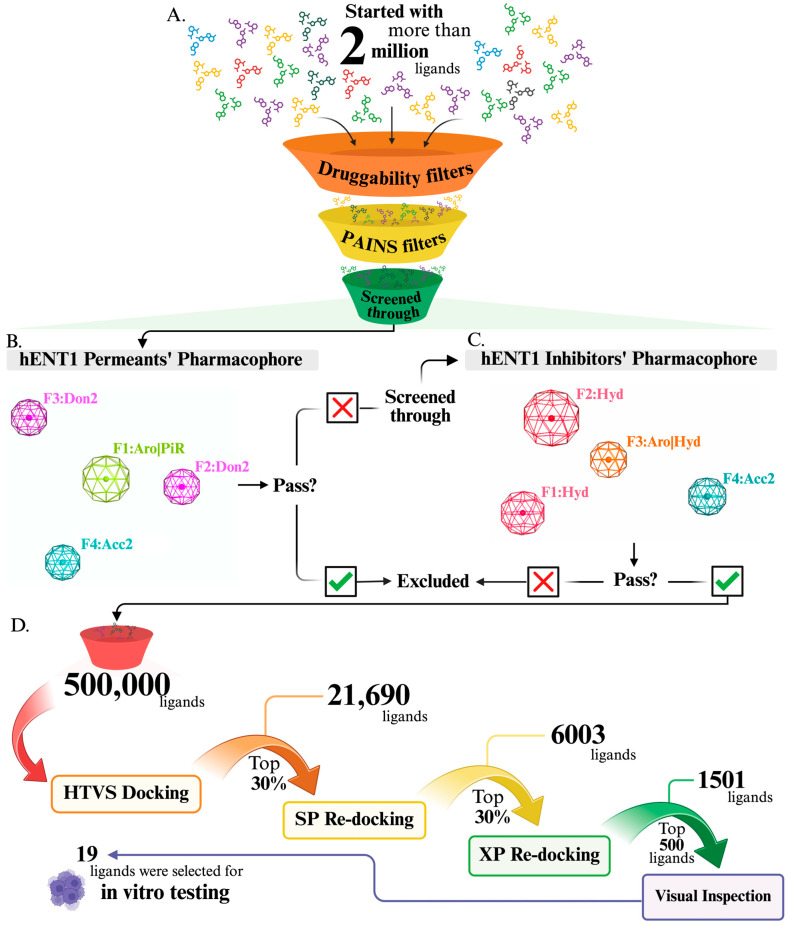
Hybrid ligand-based and structure-based virtual screening workflow for identifying high-affinity hENT1 inhibitors. (**A**) More than two million compounds from four commercial libraries (TimTec, ChemDiv, ChemBridge, and Enamine) were sequentially filtered using drug-likeness criteria (Lipinski’s and Veber’s rules) and PAINS removal. (**B**) A validated hENT1 permeant pharmacophore filter was applied to exclude permeant-like molecules. (**C**) A validated hENT1 inhibitor pharmacophore filter was then used to retain potential inhibitors. (**D**) Approximately 500,000 compounds underwent successive docking stages (HTVS, SP, and XP, respectively), followed by visual inspection to identify the top hits. This workflow efficiently narrowed the library to 19 high-affinity, drug-like hENT1 inhibitors suitable for experimental validation.

To identify potential hENT1 inhibitors, a combined structure-based and ligand-based virtual screening approach was employed. In our previous study [27], a comprehensive literature review was conducted to compile all known inhibitors and substrates, which were then used to generate and validate pharmacophore queries for each of them, resulting in two validated pharmacophore models—one for inhibitors and the other for permeants. The inhibitor pharmacophore showed high selectivity and specificity, with rates of 92% and 88%, respectively. Furthermore, the model was shown to successfully predict and identify known hENT1 inhibitors [27]. In the current study, a dataset exceeding 2 million compounds was compiled for virtual screening, as shown in Figure 1. These compounds were obtained from commercially available ligand databases: TimTec, ChemDiv, ChemBridge, and Enamine [28,29,30,31].

As an initial step, the dataset was filtered based on drug-likeness criteria to ensure that ligands exhibited physicochemical properties that would make them suitable for oral bioavailability, where failure to meet these criteria might result in poor absorption [32]. Employing Lipinski’s and Veber’s rules [32,33], ligands that did not meet the standard thresholds for these rules were excluded: hydrogen bond acceptors (HBA) ≤ 10, hydrogen bond donors (HBD) ≤ 5, molecular weight (M.W.) ≤ 500, LogP ≤ 5.0, rotatable bonds (RTB) ≤ 10, and topological polar surface area (TPSA) ≤ 140. To prevent any false-positive results in virtual screening, the dataset was further filtered using the PAINS-Remover filter and promiscuity filters [34] (Figure 1A). The compounds were then prepared using the LigPrep module in Maestro software (Schrödinger, 2024) [35] to form all possible tautomers and stereoisomers. A ligand-based virtual screening was performed on the prepared ligand library, which was screened against our validated pharmacophore models to exclude permeant-like compounds and retain inhibitor-like ligands [27] (Figure 1B,C). Ultimately, the library was downsized to approximately 500,000 ligands.

As shown in Figure 1D, a structure-based virtual screening was initiated on the final ligand library, where three consecutive stages of the docking process were applied on the library within a previously prepared active site: high-throughput virtual screening (HTVS), standard precision (SP), and extra precision (XP). The precision and accuracy improved with each stage; consequently, computational time increased in each subsequent step. The top 30% of the docked compounds from the HTVS output were redocked using SP, and subsequently the top 30% of its output were re-docked using the XP mode. This docking process reduced the library to 1501 ligands. The tautomers were then removed, retaining the top-ranked conformation for each ligand to further minimize the list for ease of selection.

The ligands were ranked based on their docking scores; the more negative the docking score, the better the binding affinity, predicting a higher affinity and inhibition of the transporter. The lowest-scored ligands were visually inspected within the active site to identify compounds demonstrating good fitting and effective interactions with the transporter using the molecular visualization software MOE (MOE, 2023.02) [36]. These shortlisted compounds were further evaluated using SwissADME to assess their predicted potential as viable lead candidates for future development [37]. Additionally, the compounds were screened for small colloidally aggregating molecules (SCAMs) using the boosted aggregation detection (BAD) filter to minimize the likelihood of false positives [38]. Ultimately, 19 compounds (Supplementary Table S1) were ordered from ChemDiv (San Diego, CA, USA) and experimentally tested, as detailed in the following sections.

### 2.2. Establishment of a Gemcitabine-Based Functional Cell Assay for Evaluating Inhibition of hENT Transport

Before evaluating the 19 compounds for their potential transport-inhibitory effects, it was necessary to establish a reliable cell-based assay to assess hENT transport activity. The assay design was based on the principle that gemcitabine (GEM), a nucleoside analog and widely used anticancer drug, enters cancer cells primarily via hENT1 [39]. GEM cytotoxicity can thus serve as a functional readout of transport activity in cancer cells (Supplementary Figure S1). To characterize this relationship, cells were incubated with a range of GEM concentrations (0.001–10,000 µM). GEM cytotoxicity was assessed using the MTT assay after 48 h of treatment. Subsequently, dose–response curves and IC50 values were determined by non-linear regression analysis.

**Figure 2:**
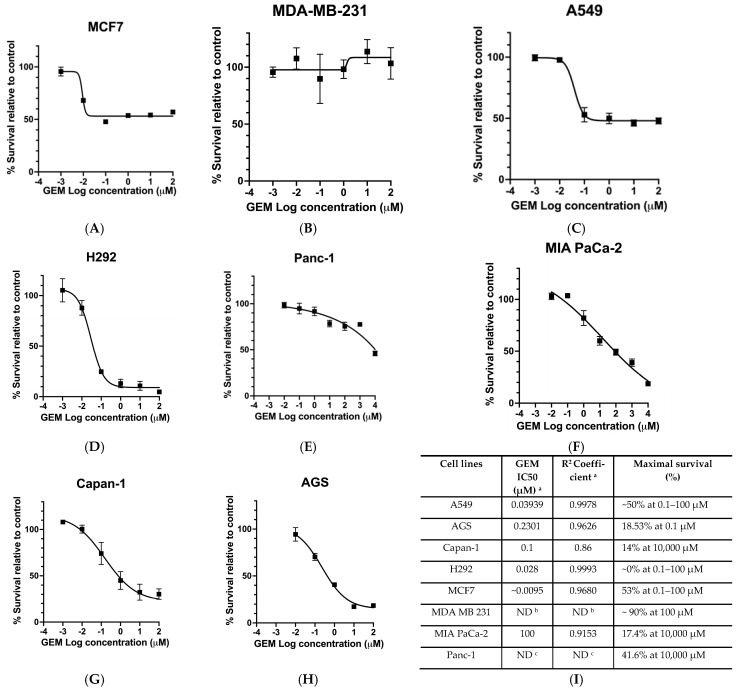
GEM reduced cancer cell survival in multiple cancer cell lines in a concentration-dependent manner. (**A**–**H**) Dose–response curve for breast (MCF7, MDA MB 231), lung (A549, H292), pancreatic (Panc-1, MIA PaCa-2, Capan-1) and stomach (AGS) cancer cell lines after treatment with Gemcitabine (GEM) for 48 h. (**I**) Calculated GEM IC50 values for the various cancer cell lines. Data points are represented as means ± SEMs; *n* = 3. ^a^ IC50 was calculated using non-linear regression (R) from cell survival dose–response curves. ^b^ ND: Not determined due to the minimal toxicity of GEM to MDA MB 231. ^c^ ND: Not determined due to GEM’s moderate cytotoxicity at 10,000 µM.

To identify a suitable cell line for developing the cell-based assay to screen for inhibitors, we first evaluated the sensitivity of eight human cancer cell lines to GEM. The cell lines tested included H292, A549 (lung cancer), MCF7, MDA-MB-231 (breast cancer), Capan-1, Panc-1, MIA PaCa-2 (pancreatic cancer), and AGS (gastric cancer). We found that GEM sensitivity varied considerably among the eight cell lines tested (Figure 2A–I). H292 lung cancer cells exhibited high sensitivity, with an IC50 of 28 nM (Figure 2D,I). Other sensitive cell lines included MCF7 (IC50 = 9 nM; Figure 2A,I) and A549 (IC50 = 40 nM; Figure 2C,I). In contrast, AGS, with an IC50 of 0.23 µM (Figure 2H,I), and Capan-1, with an IC50 of 0.1 µM (Figure 2G,I), showed moderate sensitivity. Panc-1 (Figure 2E,I), MDA-MB-231 (Figure 2B,I), and MIA PaCa-2 (Figure 2F,I) were largely resistant to GEM at concentrations up to 100 µM. Hence, the five cell lines—H292, MCF7, A549, AGS, and Capan-1—that displayed high to moderate GEM sensitivity were used for subsequent experiments.

To assess whether GEM sensitivity correlated with hENT1 expression, the baseline mRNA expressions of hENT1 in the eight cancer cell lines were determined using the mRNA database in the Human Protein Atlas (www.proteinatlas.org, accessed on 3 November 2023) (Supplementary Figure S2). Interestingly, we found that Panc-1 and MDA-MB-231 cells, despite showing the highest relative hENT1 mRNA expression (Supplementary Figure S2), exhibited no GEM cytotoxicity, even at high GEM concentrations (Figure 2I). By contrast, H292 cells, with the lowest hENT1 mRNA expression (Supplementary Figure S2) among the tested lines, were highly sensitive to GEM, with an IC50 of 28 nM (Figure 2I). This inverse correlation between hENT1 mRNA expression and GEM sensitivity indicates that mRNA levels alone do not predict functional transport activity, highlighting a critical limitation of transcript-based biomarkers.

All five other cancer cell lines showed medium hENT1 mRNA expression. Low expression of hENT2 and very low expression of hENT3 and hENT4 were observed (Supplementary Figure S2), whereas hCNT1, 2, and 3 were not detectable in any of the cell lines (www.proteinatlas.org, accessed on 3 November 2023). The low hENT2 expression across all tested cell lines, including H292, suggests that hENT1 is likely the predominant functional nucleoside transporter in these cells.

**Figure 3:**
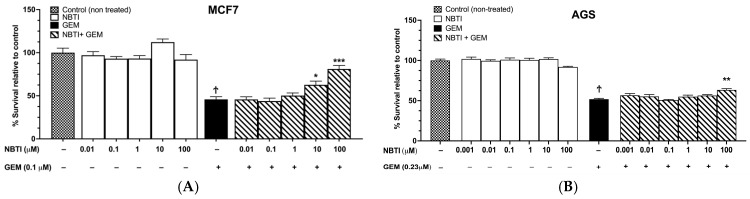

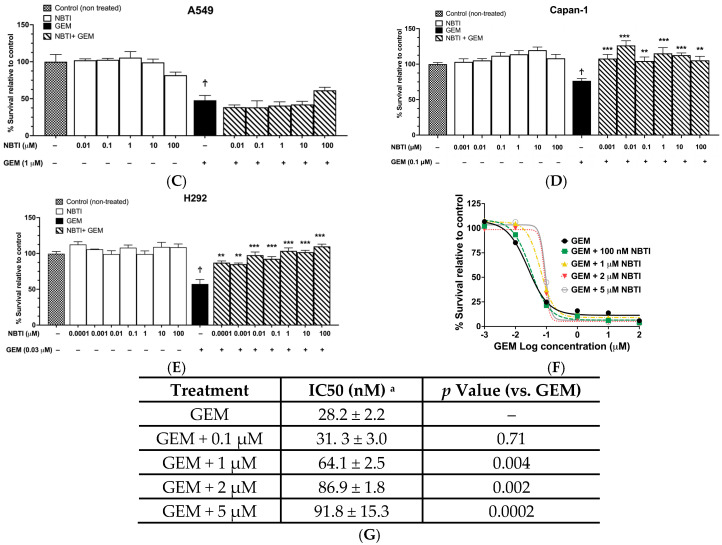
NBTI blocked GEM-induced cytotoxicity in a subset of cancer cell lines. 4-nitrobenzylthioinosine (NBTI)’s effect on GEM-induced cytotoxicity in five cancer cell lines: (**A**) MCF7, (**B**) AGS, (**C**) A549, (**D**) Capan-1 and (**E**) H292. (**F**) Increasing NBTI concentrations shifted the GEM dose–response curve to the right, increasing GEM IC50 values in H292 cells (**G**). ^a^ IC50 values were calculated using non-linear regression. Data represent means ± SEMs; *n* = 3–5; ^Ϯ^ *p* < 0.001 GEM vs. control; * *p* < 0.05, ** *p* < 0.01, *** *p* < 0.001 vs. GEM-treated cells.

To determine which of the selected cell lines showed hENT1-dependent GEM cytotoxicity, we next tested whether pharmacological inhibition of the transporter could block GEM-induced cytotoxicity in the five selected cell lines (MCF7, A549, AGS, H292, and Capan-1) (Figure 3). NBTI, a potent and selective hENT1 inhibitor at nanomolar concentrations, was used for this purpose. NBTI alone showed no cytotoxicity in any cell line across a wide range of concentrations from nanomolar to 100 µM concentrations (Figure 3A–E). GEM-induced cytotoxicity was then assessed by measuring IC50 values following co-treatment with the same range of nano- to micromolar NBTI concentrations. NBTI did not block GEM-induced cytotoxicity in A549 cells (Figure 3C). In MCF7 and AGS cells, inhibition was observed only at high concentrations (≥10 µM) (Figure 3A,B), where NBTI may lose selectivity for hENT1. In Capan-1 cells, NBTI blocked GEM-induced cytotoxicity, but the effect was not concentration-dependent (Figure 3D), suggesting that maximal inhibition was reached or that other minor transport mechanisms or resistance pathways were involved. In contrast, H292 cells were the only cell line to show a clear, concentration-dependent inhibition of GEM-induced cytotoxicity at nanomolar concentrations (0.1–100 nM), where NBTI selectively inhibits hENT1, up to 10 µM, where it inhibits hENT1 and hENT2 (Figure 3E). Based on these findings, H292 cells were selected as the primary assay model, as they demonstrated robust, concentration-dependent, hENT-dependent GEM uptake, making them ideal for evaluating inhibitors.

To establish a reference assay as a benchmark for testing the 19 candidate inhibitors, we generated GEM dose–response curves in H292 cells in the presence of increasing concentrations of NBTI (Figure 3F). H292 cells were pre-incubated with NBTI (100 nM, 1 µM, 2 µM, or 5 µM) for 2 h, then treated with GEM (0.001–100 µM) for 48 h. NBTI at 100 nM produced no significant shift in GEM IC50 (31.3 ± 3 nM vs. 28.2 ± 2.2 nM for GEM alone, *p* = 0.71; Figure 3G), indicating that this concentration was insufficient to block hENT1-mediated GEM uptake and hence necessitated testing higher NBTI concentrations. At 1 µM, 2 µM, and 5 µM, NBTI produced concentration-dependent increases in GEM IC50: 64.1 ± 2.5 nM (*p* = 0.004), 86.9 ± 1.8 nM (*p* = 0.002), and 91.8 ± 15.3 nM (*p* = 0.0002), respectively, relative to the GEM IC50 of 28.2 ± 2.2 nM (Figure 3G). Therefore, concentrations of 1–5 µM for NBTI were selected for evaluating candidate inhibitors, as this range produced measurable, concentration-dependent effects on GEM sensitivity while remaining below concentrations that might cause off-target effects. This concentration-dependent shift was used as a reference framework, and the inhibitory effect of each candidate compound was evaluated by comparing its ability to alter GEM IC50 under the same conditions.

Given that H292 cells (1) demonstrated high GEM sensitivity (IC50 = 28.2 nM), providing a sensitive readout for detecting transport inhibition (2), robust, concentration-dependent protection from GEM cytotoxicity was shown by NBTI, confirming that GEM-induced cell death is mediated by nucleoside transporter-dependent uptake, even at nanomolar concentrations known to inhibit only hENT1, and (3) the clear dose–response relationship with NBTI allows quantitative comparison of inhibitor potencies, indicate that H292 cells represent an appropriate functional assay system for ENT inhibitor screening.

### 2.3. Cytotoxicity Assessment of the Nineteen Candidate Compounds Determined That Putative hENT1 Inhibitors Were Not Cytotoxic to Fibroblasts and H292 Cells

**Figure 4:**
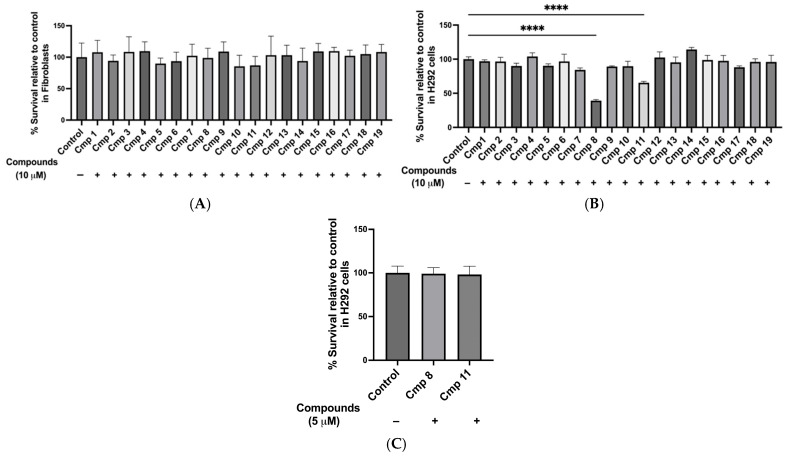
The nineteen putative hENT1 inhibitors were non-cytotoxic to fibroblasts and H292 model cells. (**A**) Cytotoxicity testing on fibroblasts (**A**) and H292 (**B**) at 10 µM. Compound (Cmp) **8** and Cmp **11** re-tested at 5 µM (**C**). Data are presented as means ± SDs; *n* = 3–4; **** *p* < 0.0001.

Before screening the 19 candidate compounds for inhibitory activity, we evaluated their safety profiles in normal cells. The compounds were tested at 10 µM on NIH 3T3 mouse fibroblasts (a non-transformed cell line commonly used to assess selectivity and general cytotoxicity). In NIH 3T3 fibroblasts, all 19 compounds were non-cytotoxic at 10 µM, with cell viabilities ranging from 95% to 105% (ns; *p* > 0.99) compared to the DMSO vehicle control (Figure 4A). The findings suggest a favorable safety profile in normal cells.

We then examined compound safety in the H292 cell line, the model system used for the transport inhibition assay, to ensure that the compounds themselves did not compromise cell viability via compound-mediated toxicity. At 10 µM, 17 out of the 19 inhibitors showed no significant cytotoxicity compared with untreated H292 control cells (ns; *p* > 0.99, compound **7** *p* = 0.37; Figure 4B). Compounds **8** and **11**, however, exhibited significant cytotoxicity at this concentration (cell viability: 58.4 ± 4.8% for compound **8** and 65.2 ± 6.5% for compound **11**, *p* < 0.0001) and were therefore re-tested at 5 µM, where cell viability was restored to 99.1 ± 6.3% for compound **8** and 98.1 ± 8.7% for compound **11** (ns; *p* > 0.99 vs. untreated controls at 100 ± 7.7%; Figure 4C). Accordingly, all compounds were tested at 10 µM in the transport inhibition assay, except for compounds **8** and **11**, which were tested at 5 µM.

In summary, the cytotoxicity results showed that 17 out of 19 compounds were safe at 10 µM in both normal and H292 cells, so they could be used in the screening assay at this dose. Compounds **8** and **11** were toxic at 10 µM in H292 cells but could still be tested at lower concentrations, such as 5 µM or less.

### 2.4. Screening and Characterization of Compounds with Inhibitory Activity

#### 2.4.1. Seven Putative Inhibitors Exhibited Similar hENT Inhibition to NBTI at 10 µM

**Figure 5:**
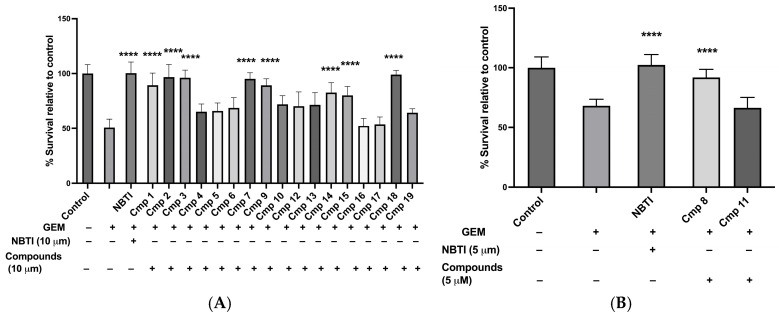
Nineteen candidate compounds were tested for their ability to block GEM-induced cytotoxicity in H292 cells. (**A**) Compounds tested at 10 µM. (**B**) Compounds **8** and **11** tested at 5 µM. Data are presented as means ± SDs; *n* = 3–5; **** *p* < 0.0001 vs. GEM-treated cells.

Next, we evaluated the 19 putative inhibitors using the transport inhibition assay. Each compound, including NBTI as a positive control, was tested at 10 µM (Figure 5A), except compounds **8** and **11**, which were tested at 5 µM due to cytotoxicity at 10 µM (Figure 5B). This initial screening concentration (10 µM) was selected to maximize the likelihood of detecting inhibitory activity; compounds showing efficacy at this concentration would then be tested at lower concentrations (1, 2, and 5 µM) in dose–response experiments in the upcoming set of experiments to determine their IC50 values and assess their potency. During this initial screening, H292 cells were pre-incubated with each compound for 2 h, then co-treated with 28 nM GEM (the previously determined GEM IC50 for H292) for 48 h. Using GEM at the IC50 concentration provides an optimal dynamic range for detecting both increases (indicating inhibition of hENT-mediated GEM uptake) and decreases (indicating enhanced uptake or other effects) in cell viability. Cell viability was assessed using MTT assays.

The positive control, NBTI 10 µM, effectively blocked GEM-induced cytotoxicity, increasing cell viability to 100.3 ± 10% compared to 50.7 ± 7.8% with GEM alone (*p* < 0.0001; Figure 5A). Compounds that showed protective effects comparable to NBTI were prioritized for further study. Nine of the nineteen compounds increased cell viability relative to cells treated with GEM alone. Specifically, compounds **1**, **2**, **3**, **7**, **9**, and **18** showed significant increases in cell viability (ranging from 89.3% to 99%, *p* < 0.0001; Figure 5A), indicating effective inhibition of hENT-mediated GEM uptake. Compounds **14** and **15** also showed a significant effect in blocking GEM-induced cytotoxicity (82.5 ± 9.1% and 80.0 ± 8.4% viability, respectively; *p* < 0.0001), but their protective activity was weaker than that of the other compounds and less comparable to NBTI, therefore, they were not included in further testing. Among the compounds tested at 5 µM, compound **8** enhanced cell viability in GEM-treated cells to 91.8 ± 6.8% (*p* < 0.0001), whereas compound **11** did not (66.5 ± 8.7%, ns; *p >* 0.99; Figure 5B). Based on these results, we proceeded to dose–response experiments with seven compounds that effectively blocked GEM-induced cytotoxicity: 1, 2, 3, 7, 8, 9, and 18.

#### 2.4.2. Compounds **2** and **3** Displayed High Potency in Inhibiting hENT Transport at 2 and 5 µM

As previously demonstrated, a reference assay was established using NBTI as a benchmark, where 1, 2, and 5 µM NBTI resulted in rightward shifts of GEM dose–response curves (GEM: 0.1–100 µM), yielding IC50 values of 64.1, 86.9, and 91.8 nM, respectively, compared to 28.2 nM for GEM alone (Figure 3G). These IC50 values serve as benchmarks for comparing candidate inhibitors. Using the same experimental setup, we generated GEM dose–response curves (GEM: 0.1–100 µM) in the presence of each of the seven lead compounds (**1**, **2**, **3**, **7**, **8**, **9**, and **18**) at concentrations of 1, 2, and 5 µM. From these curves, GEM IC50 values were determined for each compound at each concentration. To allow direct and reliable comparison of inhibitory potency across compounds and concentrations, the fold-change in GEM IC50 was calculated relative to cells treated with GEM alone in all experiments.

**Figure 6:**
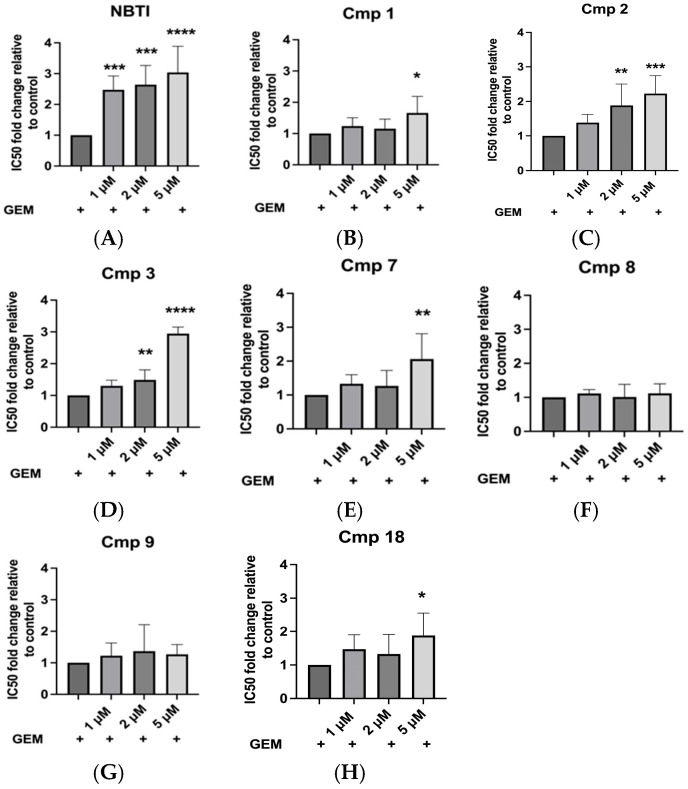
Fold-changes in GEM IC50 values demonstrate concentration-dependent hENT inhibition by selected compounds. Fold-changes in GEM IC50 values for (**A**) NBTI and (**B**–**H**) compounds **1**, **2**, **3**, **7**, **8**, **9**, and **18** at different concentrations. Data represent means ± SDs; *n* = 6; * *p* < 0.05; ** *p* < 0.01, *** *p* < 0.001, **** *p* < 0.0001 vs. GEM-only control.

NBTI produced fold-changes of 2.5 ± 0.4 at 1 µM, 2.6 ± 0.6 at 2 µM, and 3.0 ± 0.9 at 5 µM (*p* = 0.0008, *p* = 0.0004, and *p* < 0.0001, respectively; Figure 6A). Compounds **8** and **9** did not affect the GEM IC50 at any of the concentrations tested (ns; *p* > 0.99, compound **9** *p* = 0.62 at 2 µM; Figure 6F,G), indicating that they do not effectively inhibit hENT-mediated GEM uptake under these conditions. In contrast, compounds **1**, **7**, and **18** showed a significant increase in fold-change, but only at 5 µM (fold-changes: 1.7 ± 0.5, 2.1 ± 0.7, and 1.9 ± 0.7; *p* = 0.012, *p* = 0.003, and *p* = 0.018, respectively), not at lower concentrations (1 or 2 µM, *p* > 0.05; Figure 6B,E,H), suggesting moderate inhibitory activity. Compounds **2** and **3** showed significant increases in the fold-change of GEM IC50 at 2 µM (1.9 ± 0.6 and 1.5 ± 0.3, respectively; *p* = 0.006 and *p* = 0.0024) and 5 µM (2.2 ± 0.5 and 2.9 ± 0.2, respectively; *p* = 0.0003 and *p* < 0.0001; Figure 6C,D). These results indicate that compounds **2** and **3** exhibit functional activity consistent with hENT inhibition, with fold-changes approaching those observed for NBTI, particularly at higher concentrations. Hence, compounds **2** and **3** could represent promising agents with demonstrated functional activity in this cell-based assay.

### 2.5. Protein–Ligand Interaction Fingerprints, Docking Score Comparisons, and Binding Mode Analysis

**Figure 7:**
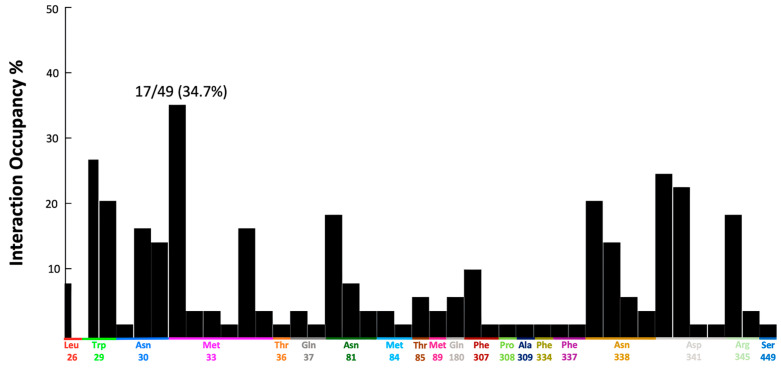
Protein–ligand interaction fingerprints (PLIF) analysis of 49 known hENT1 inhibitors showing the frequency of interactions with key active-site residues.

For a more comprehensive understanding of inhibitors’ affinity and efficacy, protein–ligand interactions were analyzed. Therefore, these interactions were carefully analyzed in the post-docking stage. Key active site residues were identified based on a thorough review of the literature. A protein–ligand interaction fingerprint (PLIF) analysis was then performed on the reported known inhibitors (Figure 7). The resulting interaction profiles were used to compare our top-scoring hits from biological assays with the reference inhibitor, NBTI.

PLIF analysis showed that most of the ligands interacted with the key residue Met33 with 34% involvement. Trp29, Asn338, Asp341, and Arg345 were also identified as interacting residues (Figure 7). In line with previous studies, Trp29 has been reported to be a key residue that interacts with the natural inhibitor dilazep and its derivatives [23]. Similarly, Phe307, Asn338, and Gln189 have also been identified as key bioactive residues [40,41,42].

**Figure 8:**
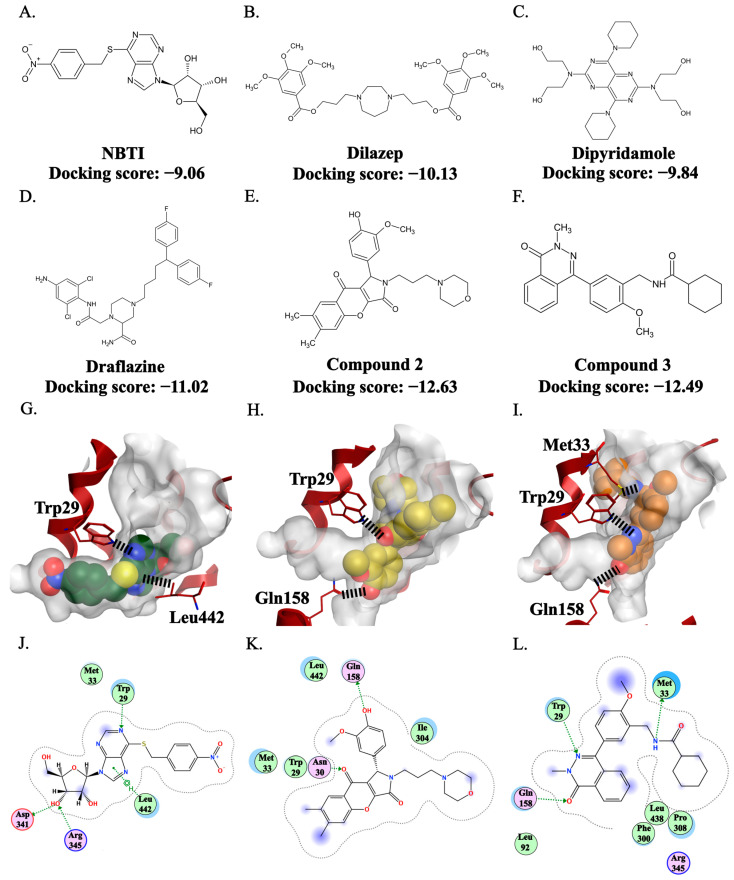
Comparative docking analysis with known hENT1 inhibitors and the binding modes of NBTI, compound **2**, and compound **3**. The chemical structures and corresponding docking scores (kcal/mol) for NBTI (**A**), dilazep (**B**), dipyridamole (**C**), draflazine (**D**), compound **2** (**E**), and compound **3** (**F**). Docking poses of NBTI (**G**), compound **2** (**H**), and compound **3** (**I**) within the hENT1 binding pocket. Two-dimensional interaction maps showing ligand–residue interactions for NBTI (**J**), compound **2** (**K**), and compound **3** (**L**).

Additionally, a comparative docking analysis was performed, including reported inhibitors like NBTI, Dilazep, Dipyridamole, and Draflazine. As shown in Figure 8E,F, compounds **2** and **3** exhibited more favorable docking scores (−12.63 and −12.49 kcal/mol, respectively) compared to the reference inhibitor, NBTI (−9.06 kcal/mol) (Figure 8A), and the other inhibitors, Dilazep, Dipyridamole, and Draflazine (Figure 8B–D). This supports that compounds **2** and **3** possess competitive binding potential within the active site.

The reference inhibitor, NBTI, was shown to block the transporter in a horizontal orientation, forming four hydrogen bonds and hydrophobic interactions with surrounding residues, including the key residue Trp29 (Figure 8G,J). In contrast, compounds **2** and **3** demonstrated vertical blocking of the transporter (Figure 8H,I), resulting in fewer overall interactions. Compound **2** formed two interactions, while compound **3** formed three (Figure 8K,L). However, both compounds retained the interaction with the key residue Trp29. Interestingly, the loss of some interactions was compensated by new ones; for example, compound **3** formed an interaction with Met33, the most frequently involved residue among the known inhibitors in our PLIF study. This may explain why compound **3** exhibited activity similar to NBTI. Furthermore, the vertical orientation of these compounds enabled them to access and fill new binding regions that NBTI did not reach.

### 2.6. Assessment of Predicted Pharmacokinetic and Drug-like Characteristics

The pharmacokinetic properties, drug-likeness, and promiscuous binding potential of compounds **2** and **3** were evaluated using the SwissADME and BAD tools, with NBTI as a reference [37,38]. A full assessment of the hits is presented in Table 1. Both compounds **2** and **3** were predicted to have high gastrointestinal (GI) absorption, which aligns with the initial filtration based on drug-likeness criteria, such as Lipinski’s rule of five and Veber’s rules [32,33]. In contrast, NBTI displayed poor predicted GI absorption. Accordingly, both compounds met other drug-likeness criteria evaluated by SwissADME, including Ghose’s, Egan’s, and Muegge’s rules.

Compound **3** was predicted to have the ability to cross the blood–brain barrier (BBB), unlike compound **2**. However, since compound **3** is not predicted to bind to the P-glycoprotein (P-gp) efflux pump, there may be a risk of brain accumulation, as the compound might not be efficiently transported out of the brain. Both compounds were predicted to interact with the cytochrome P450 family of enzymes and, hence, to cause drug–drug interactions. No compounds had PAINS substructures. This was anticipated as PAINS filtering was applied during the dataset preparation stage. However, NBTI violated Brenk’s rules, which indicated the presence of potentially reactive fragments. These reactive fragments may account for the off-target activities reported in the literature, since they have the potential to interact indiscriminately with biological sites not meant to be targeted.

All compounds were evaluated using the BAD tool, which screens for SCAMs to assess for aggregation potential. Neither compound **2** nor compound **3** was flagged as a potential aggregator, and no similar aggregators were found in the literature.

## 3. Discussion

Current hENT1 inhibitors have a poor pharmacological profile, underscoring the need for new hENT1 inhibitors. We developed and validated a non-radioactive, GEM-based functional assay using H292 cancer cells to assess the inhibitory activity of 19 candidate compounds identified through a dual-pharmacophore virtual screening protocol. We found that compounds **2** and **3** were good leads, as they inhibited GEM-mediated cell cytotoxicity to levels close to NBTI. They also exhibited higher docking scores than NBTI and a good ADME profile and interacted with residues Trp29 and Met33 within the hENT1 core cavity. These are key residues that have previously been shown to interact with well-established hENT1 inhibitors.

H292 cells proved to be the optimal cell line for the ENT inhibitor’s validation. This was due to two factors. First, H292 demonstrated high sensitivity to gemcitabine (IC50 = 28 nM), consistent with another study reporting an IC50 of 3 nM for GEM-mediated cytotoxicity after a 72 h incubation period [43]. Second, H292 was the only cell line that showed a clear dose-dependent inhibition of GEM-mediated cytotoxicity at nanomolar to micromolar concentrations of the potent hENT1 inhibitor, NBTI.

An important observed anomaly was that, despite low hENT1 transcript expression in H292 cells, sensitivity to GEM was high (IC50 = 28 nM), whereas in the Panc-1, MiaPaCa-2, and MDA-MB-231 cell lines with high hENT1 transcript expression, GEM resistance was observed. This is consistent with the literature, which reports that the pancreatic cancer cell lines BxPC-3 and MIA PaCa-2 are inherently resistant. Also, MRP5, an efflux pump, was highly expressed in Panc-1 and MIA PaCa-2 cell lines, responsible for the efflux of GEM monophosphate and reduction of GEM-mediated cytotoxicity [44]. Regarding lung cancer lines, Achiwa et al. [45] showed that hENT1 and deoxycytidine kinase (dCK) were both important determinants of gemcitabine-mediated cytotoxicity across 22 NSCLC cell lines. High GEM sensitivity despite low hENT1 expression in H292 could be explained by the fact that the functional activity of transporters such as hENT1 is not solely determined by transcription level but primarily by post-transcriptional regulation, protein stability, and membrane trafficking [5,46]. Furthermore, GEM-mediated cytotoxicity is influenced by many factors other than hENT1 expression: (1) activation of the enzyme deoxycytidine kinase (dCK) [47], (2) inactivation of the enzyme cytidine deaminase (CDA) [48], (3) efflux pumps (MRP) [49], and (4) intrinsic DNA damage sensitivity and repair mechanisms [50]. Importantly, our NBTI validation confirmed functional hENT activity in H292 cells.

A key advantage of the GEM-based assay is its accessibility and practicality compared to traditional radioligand-based assays. The [^3^H] 2-chloroadenosine uptake assay or [^3^H]-NBMPR binding assay requires the use of radioisotopes, which are costly due to the need for specialized equipment such as a scintillation counter and waste-disposal resources, and poses safety hazards. In contrast, our validated assay employs a standard MTT assay in a 96-well plate, providing a functional readout with physiological relevance. Although the MTT assay indirectly measures hENT transport, it is highly effective for screening large numbers of compounds, unlike the direct hENT uptake assay using a radioligand. This approach enables the identification of promising compounds, which can subsequently be tested and validated using conventional radioligand-based assays.

It is worth mentioning that when using a single low dose of GEM (28 nM), nanomolar NBTI concentrations were sufficient to block GEM-mediated cytotoxicity, while with increasing concentrations of GEM these nanomolar NBTI concentrations were insufficient, necessitating a high NBTI concentration to block GEM cytotoxicity when establishing the reference assay, which likely reflects multiple factors: (1) competitive inhibition—at high gemcitabine concentrations (up to 100 µM), weak ENT transport inhibition due to low NBTI concentrations is overcome by the high GEM concentrations; (2) assay duration—our 48 h incubation allows time for compound/NBTI redistribution; and (3) the fact that indirect measurement of cytotoxicity is a cumulative product of transport, metabolism, DNA incorporation of GEM, and apoptosis.

NBTI, while highly selective for hENT1 at nanomolar–low micromolar concentrations (1 µM), selectivity may decrease at higher concentrations. However, our Human Protein Atlas analysis revealed that hENT2 expression was uniformly low across all tested cell lines, including H292, while hENT3 and hENT4 expression was very low and hCNT1-3 expression was undetectable, suggesting that hENT1 is the predominant functional nucleoside transporter in H292 cells. GEM has a higher affinity for hENT1 than for hENT2 [51], further supporting this. By extension, we postulate that the inhibitory effects of compounds **2** and **3** are likely to be mediated by hENT1, given that protein–ligand interactions showed that compounds **2** and **3** interact with previously identified key binding residues within hENT1, i.e., Trp29, and compound **3** also interacts with Met33. However, we cannot definitively exclude a contribution from hENT2; therefore, we refer to our compounds as “ENT putative inhibitors with evidence supporting predominantly hENT1-mediated activity” rather than claiming absolute hENT1 selectivity.

The distinct vertical binding orientation of compounds **2** and **3**, compared with NBTI’s horizontal orientation, represents a key finding. Both compounds retain interaction with the key residue Trp29. Compound **3** interacted with Met33, the most frequently involved residue among the known inhibitors in our PLIF study. Overall, the superior docking scores in comparison with multiple known hENT1 inhibitors, combined with key residue interactions within the hENT1 central cavity, support that compounds **2** and **3** are hENT1 inhibitors.

It is important to note that while docking scores can predict binding affinity, they do not always correlate with functional potency in cell-based assays. Compounds **2** and **3** had higher docking scores than NBTI but needed higher concentrations to show effects in functional tests. This shows the limits of computational predictions and the need for experimental validation. Docking scores estimate binding free energy based on in silico assumptions of binding affinity and binding mode under static conditions, without accounting for the protein’s molecular dynamics or the effects of inhibitors on its conformational changes. Also, the conformational transport cycle of hENT1 plays a critical role in nucleoside translocation across the cell membrane [40].

Compounds **2** and **3** exhibited a favorable ADME profile, in contrast with NBTI, which has poor GI absorption and reactive moieties. Compound **3**’s predicted ability to cross the blood–brain barrier is evidence supporting the use of hENT1 inhibitors in pain therapy [52], though the absence of P-gp substrate activity raises concerns about brain accumulation. The compounds’ interactions with CYP enzymes warrant drug–drug interaction assessment.

## 4. Study Limitations and Future Directions

There are some limitations to consider. Firstly, our cytotoxicity-based assay indirectly measures transport, so direct radiolabeled uptake assays are still needed to confirm transport inhibition. Secondly, we have not fully confirmed whether the inhibitors are selective for hENT1 over hENT2. Hence, transporter overexpression or knockdown validation, competitive transport kinetics, orthogonal biochemical confirmation, or binding force validation studies can be utilized for definitive proof. Furthermore, molecular dynamics simulations would provide valuable insight into the stability of the vertical binding mode of compounds **2** and **3** over time.

## 5. Materials and Methods

### 5.1. Protein Preparation and Grid Generation

The hENT1 co-crystal structure was obtained from the Protein Data Bank RCSB, Piscataway, NJ, USA (PDB, ID: 6OB7) [40,41]. All water molecules were removed prior to protein preparation, and then all missing residues and atoms were adjusted and corrected using the MOE protein preparation wizard (MOE, 2023.02) [36,53]. Further adjustments were employed utilizing the protein preparation wizard available in Maestro by introducing hydrogen atoms and assigning partial charges to each atom [54]. The GRIDE module within the Schrödinger Maestro was used to identify the binding site of hENT1 for docking, with the bound inhibitor serving as the centroid of the box.

### 5.2. Ligand Library Preparation

Over 2 million compounds were collected from commercially available screening libraries, including TimTec, ChemDiv, ChemBridge, and Enamine [28,29,30,31]. Veber’s rules and Lipinski’s rule of five were used to filter out compounds that did not exhibit drug-likeness characteristics [32,33]. The dataset was then curated by applying the PAINS-Remover, an online open-access server used to remove PAINS [34]. The LigPrep module in Schrödinger Maestro was used to generate all possible protonation states and tautomer forms of the ligands [55]. Later, an additional screening step was employed using our previously validated pharmacophore models [27].

### 5.3. Molecular Docking

The prepared dataset was docked into the hENT1 active site. The docking process consisted of three steps [55], and precision and accuracy increased with each step: high-throughput virtual screening (HTVS), Glide standard precision (SP), and Glide extra precision (XP) [56]. In each step, the top 30% of the docked compounds from the previous stage were re-docked using the subsequent docking mode. Duplicates of ligands were removed, retaining the highest-scoring tautomer obtained from XP docking to minimize the list for ease of selection. Visual inspection of the top docked ligands was performed to identify ligands that demonstrated optimal binding within the active site, adopting a reasonable binding orientation and showing good fitting and an absence of steric clashes or unrealistic conformations, while maintaining favorable interactions with the key residues (e.g., hydrogen bonds, hydrophobic contacts, and π–π stacking). Consequently, 19 compounds were selected for biological testing and evaluated for their pharmacokinetic and drug-like properties using SwissADME. The compounds that fulfilled all the criteria for being promising inhibitors were subsequently subjected to in vitro testing.

### 5.4. Protein–Ligand Interaction Fingerprints

All known hENT1 inhibitors reported in the literature were docked into the hENT1 binding site to identify key interacting residues. A protein–ligand interaction fingerprint (PLIF) analysis was performed using MOE, with default minimum score thresholds of 1.5 kcal/mol for hydrogen bonds and 1 kcal/mol for aromatic interactions.

### 5.5. Cell Culture

Breast cancer cell lines MCF7 (86012803) and MDA-MB-231 (92020424) and lung cancer cell lines A549 (86012804) and H292 (91091815) were obtained from ECACC (Salisbury, UK). The gastric adenocarcinoma cell line AGS (ATCC CRL1739) was a gift from Sharjah University, while the pancreatic cell lines Capan-1 (ATCC CLS-300143), PANC-1 (ATCC CRL-1469), and MIA-PaCa-2 (ATCC CRL-1420) were gifts from the Gulf Medical School (UAE). NIH 3T3 mouse fibroblasts (ATCC CRL-1658) obtained from ATCC (Manassas, VA, USA) were used for cytotoxicity assessment. All media and media supplements were purchased from Sigma-Aldrich (St. Louis, MO, USA).

A549, H292, AGS, and Capan-1 cancer cell lines were maintained in RPMI medium supplemented with 10% (*v*/*v*) FBS. PANC-1, MIA-PaCa-2, and MDA-MB-231 were grown in DMEM supplemented with 10% (*v*/*v*) FBS for PANC-1 and MIA-PaCa-2, or 15% (*v*/*v*) FBS for MDA-MB-231. MCF7 cells were cultured in MEM medium supplemented with 10% (*v*/*v*) FBS and 1% non-essential amino acids. All media were supplemented with 1% penicillin–streptomycin. Cells were maintained at 37 °C with 5% CO_2_ and sub-cultured every 2–4 days. Experiments were conducted with cells in the exponential growth phase.

### 5.6. Transcriptomic Profiling of hENT1

hENT1 transcription profiles of all cell lines were obtained from an open-access database (www.proteinatlas.org, accessed on 3 November 2023).

### 5.7. Pharmacological Treatments

The 19 potential inhibitors were obtained from the ChemDiv Commercial Library (San Diego, CA, USA). Stock solutions of the 19 potential inhibitors and NBTI (#N2255, Sigma-Aldrich, Saint Louis, MO, USA) were prepared in DMSO and subsequently diluted in RPMI culture medium containing 10% FBS and 1% penicillin–streptomycin.

### 5.8. Cytotoxicity Assays

#### 5.8.1. Establishing GEM Cytotoxicity

All assays were performed using the MTT Proliferation Assay (ab211091; Abcam, Cambridge, UK) according to the manufacturer’s instructions. Briefly, the eight cancer cell lines were seeded on 96-well plates (CL3599, Corning, MA, USA) at a density of 5.5 × 10^4^ cells/well and allowed to attach overnight. Media were then removed and replaced with fresh media with or without graded concentrations of GEM (G6423, Sigma-Aldrich, Saint Louis, MO, USA), and the plates were incubated for 48 h at 37 °C. After 48 h, 3-(4,5-dimethylthiazol-2-yl)-2,5-diphenyltetrazolium bromide (MTT) reagent was added to each well and incubated at 37 °C for ~3 h. MTT solvent was then added to each well and left overnight. The optical density (OD) in each well was measured at 590 nm using the Synergy H1 Hybrid Multi-Mode Reader (BioTek^®^ Instruments, Winooski, VT, USA). The percentage cell survival was calculated using the following formula:

% cell survival = [(OD sample − OD background)/(OD control − OD background)] × 100%

#### 5.8.2. NBTI-Mediated Inhibition of GEM-Induced Cancer Cell Cytotoxicity

Since NBTI blocks hENT1, preventing GEM from entering cancer cells and inducing toxicity, it was used to inhibit GEM transport into cancer cells and block its cytotoxicity. Cancer cells were seeded at a density of 5.5 × 10^4^ cells/well on 96-well plates and allowed to attach overnight. Media were then removed and replaced with fresh media with or without increasing concentrations of NBTI (N2255, Sigma-Aldrich, Saint Louis, MO, USA) (0.0001–100 µM). The plates were incubated for 2 h with NBTI, then media with or without GEM (28 nM) were added to each well, and the plates were incubated for a further 48 h. After 48 h, MTT reagent was added to each well, and the plates were incubated at 37 °C for 3 h. MTT solvent was then added to each well and left overnight. The OD at 590 nm in each well was determined as mentioned above.

The same protocol was used to establish a standard reference assay with four selected narrow-range NBTI concentrations (100 nM and 1, 2, and 5 µM) with or without GEM (0.001–100 µM).

#### 5.8.3. Cytotoxicity Assay on Fibroblasts

To assess the safety profile of the potential inhibitors, NIH 3T3 fibroblast cells were seeded at 5000 cells per well in 96-well plates and incubated at 37 °C in a humidified incubator. After 24 h, the original culture medium was carefully removed, and the cells were either left untreated or treated with 10 µM of each candidate inhibitor for 48 h. The subsequent procedures for assessing viability via the MTT assay followed the protocol described above.

#### 5.8.4. Cytotoxicity Assay on the H292 Model Cells

To ensure that any observed cytotoxicity was attributed solely to GEM, H292 cells were seeded at 5.5 × 10^4^ cells per well in 96-well plates and incubated at 37 °C in a humidified incubator. After 24 h, the original culture medium was removed, and the cells were either left untreated or treated with 10 µM of each candidate for 48 h. The subsequent procedures followed the MTT assay protocol described above.

#### 5.8.5. hENT Inhibition Activity Assay

To assess the activity of potential hENT1 inhibitors relative to NBTI, H292 cells were seeded at 5.5 × 10^4^ cells per well in 96-well plates and incubated at 37 °C in a humidified incubator. After 24 h, the original culture medium was carefully removed, and the cells were either left untreated or treated with 10 µM of each candidate or NBTI for 2 h. After 2 h of incubation, media with or without 28 nM GEM were added, and then the plates were incubated for 48 h. The subsequent procedures followed the protocol mentioned above.

#### 5.8.6. hENT Inhibition Potency Assay

In this experiment, H292 cells were seeded at 5.5 × 10^4^ cells per well in 96-well plates and incubated at 37 °C in a humidified incubator. After 24 h, the original culture medium was carefully removed, and the cells were either left untreated or treated with 1 µM, 2 µM, or 5 µM concentrations of each candidate compound or NBTI for 2 h. The plates were incubated for 2 h prior to adding media with or without GEM (0.0001–100 µM), and then incubated for 48 h. The subsequent procedures followed the protocol mentioned above.

### 5.9. Statistical Analysis

Data were expressed as the means ± SDs or SEMs from three to five independent experiments, as indicated in the legends. Each concentration/condition in every independent experiment was run at least in triplicate. Survival curve analysis was performed using non-linear regression analysis to generate a dose–response curve and calculate the concentration that inhibited cell survival at 50% (IC50) (GraphPad Prism version 10.0; GraphPad Software, San Diego, CA, USA). Differences between multiple means were evaluated by one-way ANOVA followed by Bonferroni’s test for multiple comparisons (GraphPad Prism version 10.0). Similarly, differences in IC50 shift between GEM and NBTI concentrations were evaluated by one-way ANOVA followed by Bonferroni’s test. A *p*-value of <0.05 was considered to indicate statistical significance.

## 6. Conclusions

In conclusion, the present study demonstrated a practical non-hazardous protocol, rather than the standard uptake assay, for screening new hENT inhibitors. Later, the top hits from the screening can be validated with a conventional radiolabeled hENT1 transporter/uptake assay. Also, we successfully integrated structure-based virtual screening with experimental validation to identify novel hENT inhibitors with favorable drug-like properties. Functional screening identified compounds **2** and **3** as exhibiting NBTI-comparable potency. These compounds are promising candidates for further development as hENT1-targeted inhibitors with potential applications in cancer chemotherapy sensitization, pain management, neurodegenerative disease, and cardiovascular protection.

## Figures and Tables

**Table 1:** Pharmacokinetic and promiscuity characteristics of compounds **2** and **3** as predicted by SwissADME and the BAD tool, compared to NBTI.

			NBTI	Cmpd2	Cmpd3
SwissADME	Pharmacokinetics	GI Absorption	Low	High	High
		BBB permeant	No	No	Yes
		P-gp substrate	Yes	Yes	No
		CYP1A2 inhibitor	No	No	No
		CYP2C19 inhibitor	No	No	Yes
		CYP2C9 inhibitor	No	Yes	Yes
		CYP2D6 inhibitor	No	Yes	Yes
		CYP3A4 inhibitor	No	Yes	Yes
		Bioavailability rate	0.55	0.55	0.55
	Medicinal Chemistry	PAINS	0	0	0
		Brenk	2	0	0
BAD Tool		SCAM	Non-aggregator	Non-aggregator	Non-aggregator

## Data Availability

Data are presented within the study.

## Supplementary Materials

The following supporting information can be downloaded at https://www.mdpi.com/article/10.3390/molecules31081293/s1, Figure S1: Principle of the GEM-Based Functional Assay for Evaluating hENT1 Activity. Figure S2: Transcriptomic expression profile of hENT and hCNT transporters in eight cancer cell lines. Table S1: The 19 potential inhibitors with their respective structure and docking scores.
