# Supplementary material for: Identification of Putative Equilibrative Nucleoside Transporter Inhibitors Through Dual-Pharmacophore Virtual Screening and Validation in a Gemcitabine-Based Cell Assay
Source: Molecules. 2026 Apr 15;31(8):1293. doi: 10.3390/molecules31081293 (PMC13118991; doi:10.3390/molecules31081293)
Supplement: Supplementary file 1 [file molecules-31-01293-s001.zip › molecules-4197895-supplementary.pdf]

A.

B.

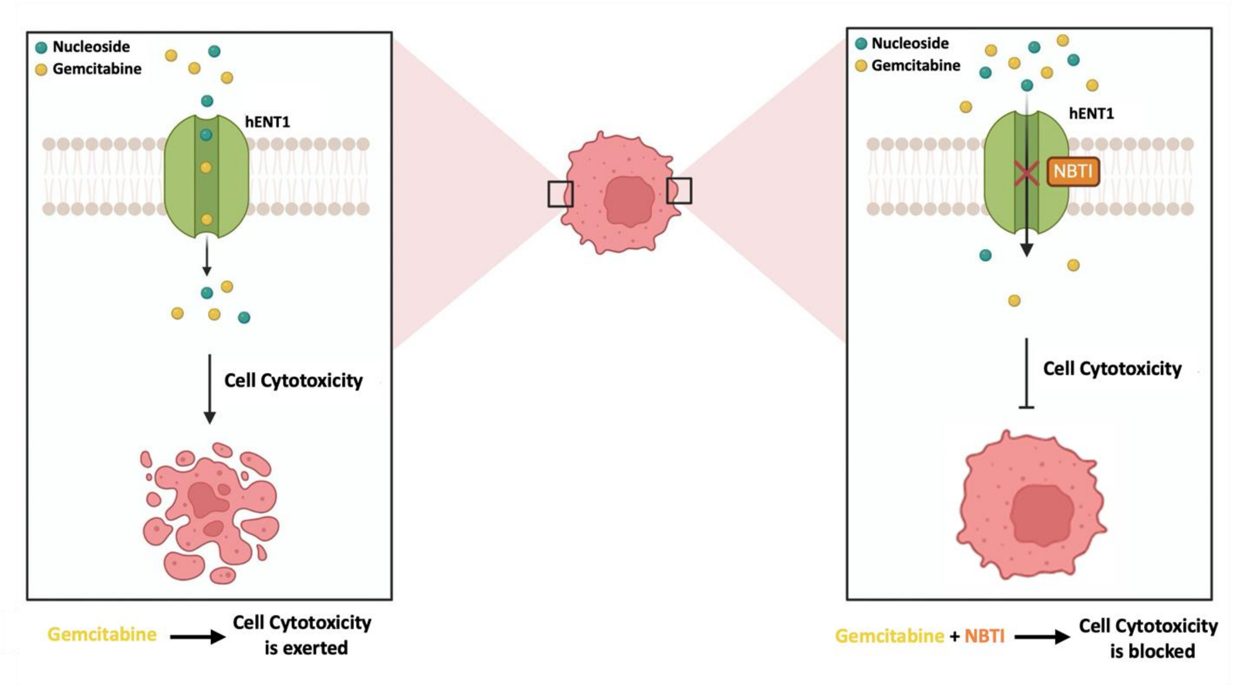

Supplementary Figure S1. Principle of the GEM-Based Functional Assay for Evaluating hENT1 Activity. (A) Gemcitabine (GEM), a nucleoside analog, enters cells primarily through the human equilibrative nucleoside transporter 1 (hENT1), resulting in intracellular accumulation and cytotoxicity. (B) In the presence of NBTI, a selective hENT1 inhibitor, GEM uptake through hENT1 is blocked, leading to reduced intracellular accumulation and decreased cytotoxicity. This assay design uses GEM-induced cytotoxicity as a functional readout of hENT1 transport activity, enabling evaluation of hENT1 inhibition in cell-based systems.

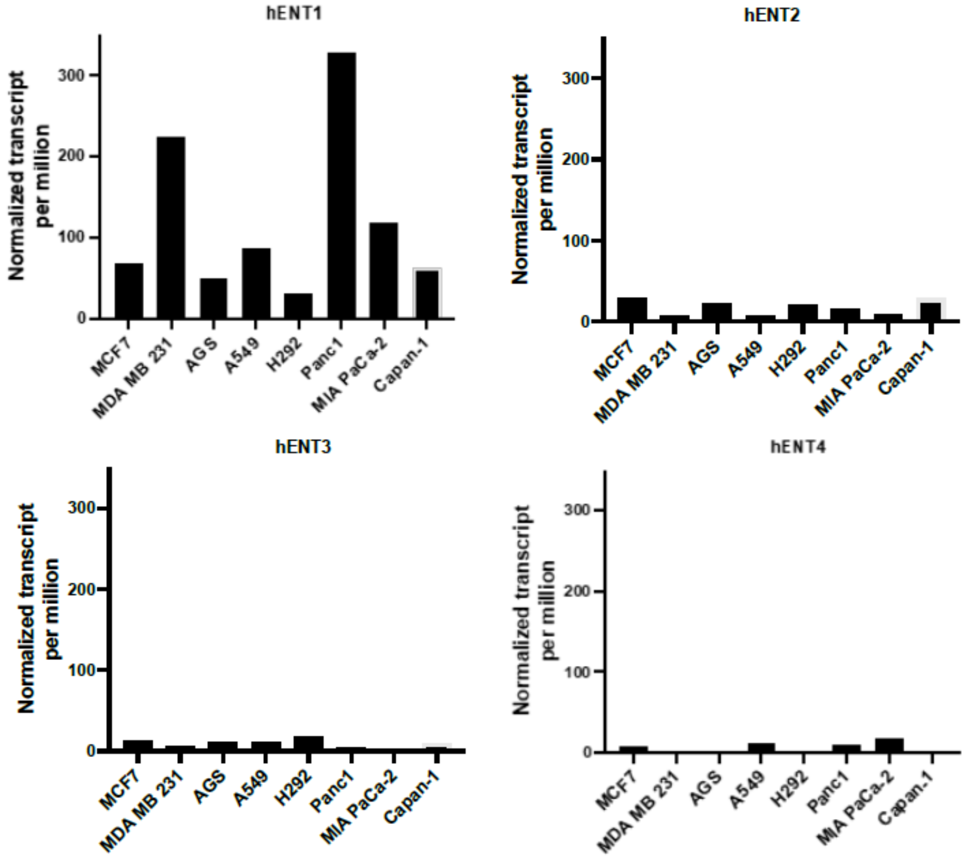

Supplementary Figure S2. Transcriptomic expression profile of hENT and hCNT transporters in eight cancer cell lines. Baseline mRNA expression levels of hENT and hCNT family members were analyzed in eight cancer cell lines—breast (MCF7, MDA-MB-231), lung (A549, H292), pancreatic (Panc-1, MIA PaCa-2, Capan-1), and gastric (AGS)—using data obtained from the Human Protein Atlas (www.proteinatlas.org). Expression levels are shown as normalized transcripts per million (nTPM). Among these cell lines, Panc-1 exhibited the highest hENT1 expression, followed by MDA-MB-231, while H292 displayed the lowest expression. The remaining lines showed intermediate hENT1 levels. hENT2 expression was low, hENT3 and hENT4 were very low, and hCNT1–3 were undetectable across all cell lines.

Supplementary Table S1. The 19 potential inhibitors with their respective structure and docking score.

Compound ID	Chemical Structure	Docking Score
**NBTI**	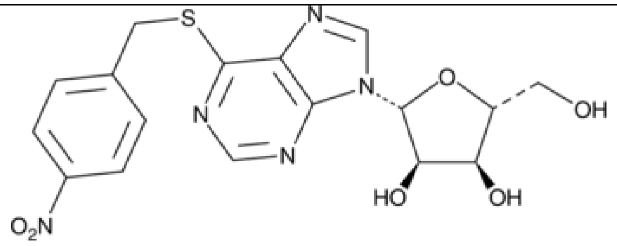	-9.06
**Cmpd 1**	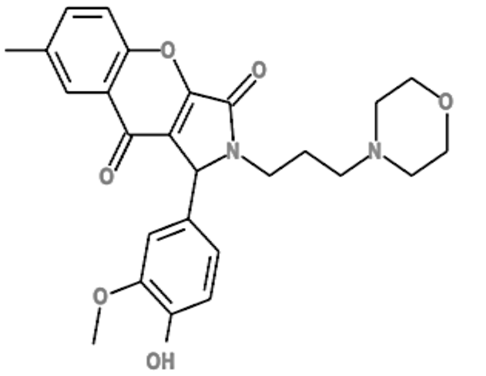	-12.10
**Cmpd 2**	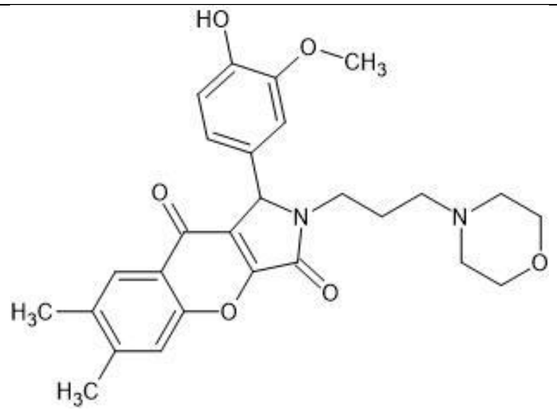	-12.63

**Cmpd 3**	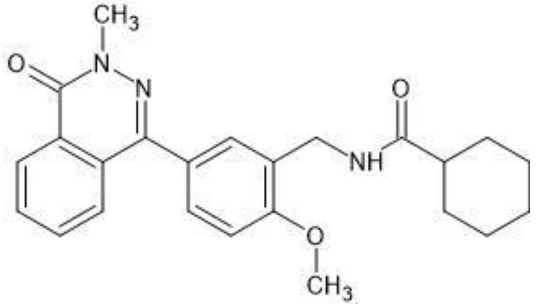	-12.49
**Cmpd 4**	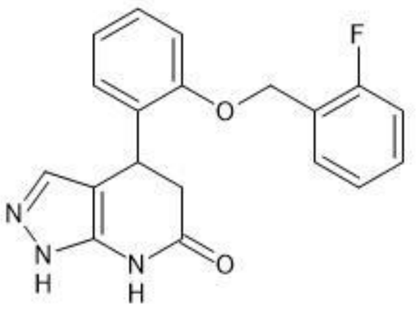	-12.47
**Cmpd 5**	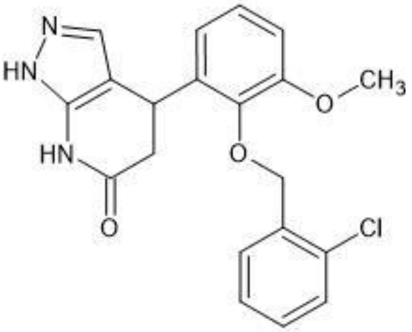	-12.51
**Cmpd 6**	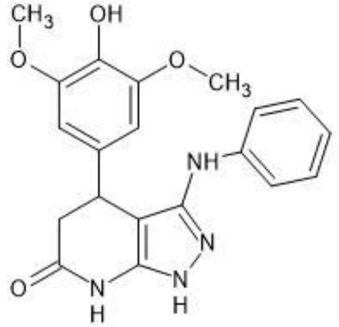	-12.63
**Cmpd 7**	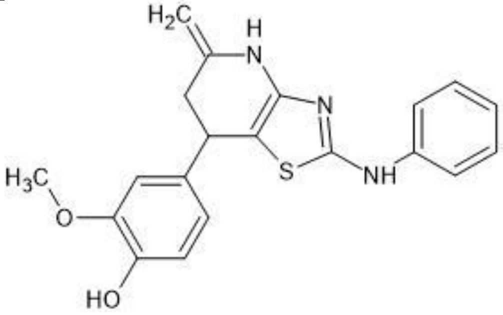	-13.12

**Cmpd 8**	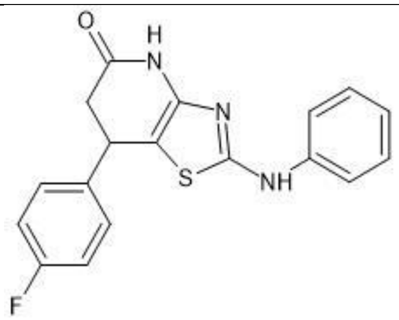	-13.36
**Cmpd 9**	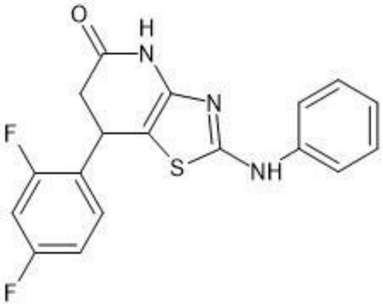	-13.44
**Cmpd 10**	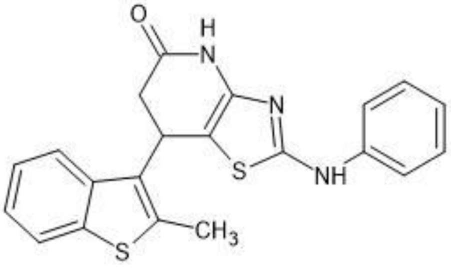	-13.72
**Cmpd 11**	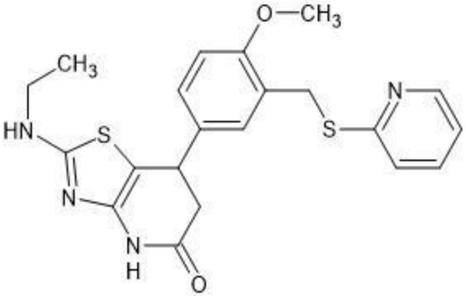	-13.29
**Cmpd 12**	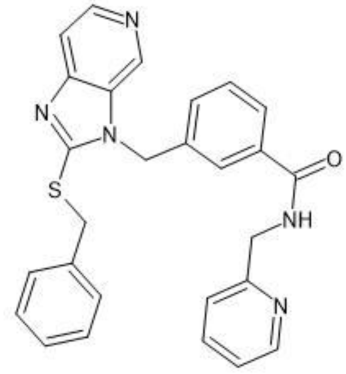	-13.26

**Cmpd 13**	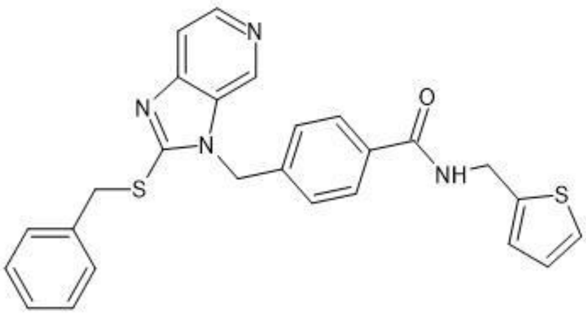	-12.55
**Cmpd 14**	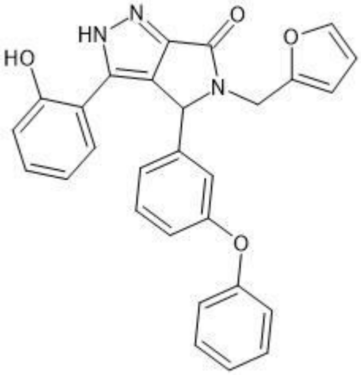	-13.00
**Cmpd 15**	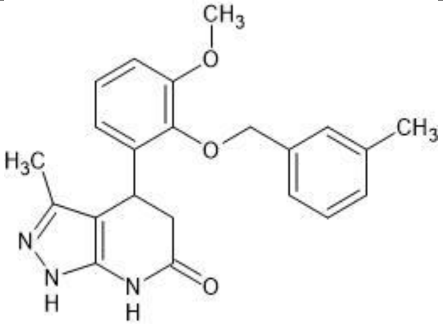	-12.57
**Cmpd 16**	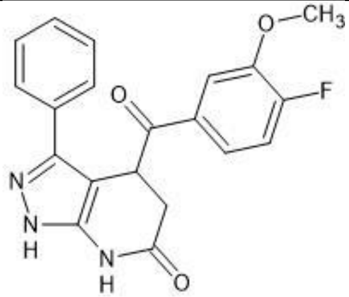	-12.63

**Cmpd 17**	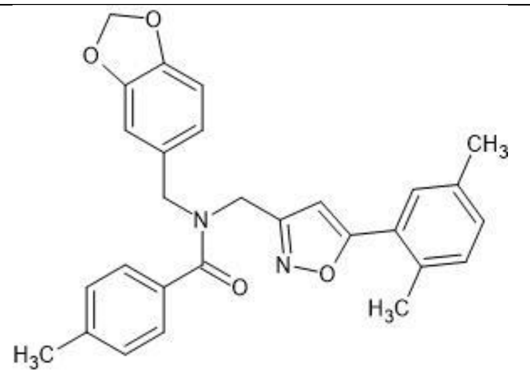	-12.61
**Cmpd 18**	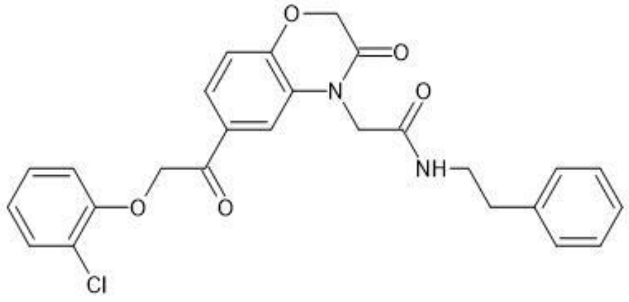	-12.47
**Cmpd 19**	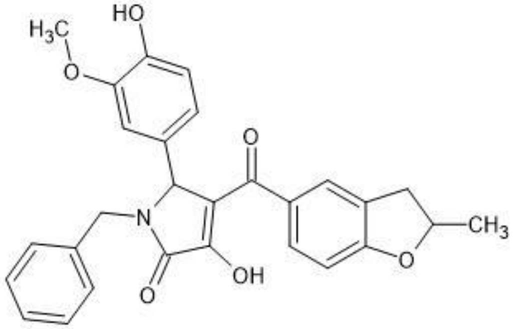	-12.63
